# Mode of delivery for confirmed macrosomia: a real-life multicentric observational study

**DOI:** 10.1186/s12884-026-09090-5

**Published:** 2026-04-27

**Authors:** Alexia Mazard, David Desseauve, Michel Boulvain, Anthony Atallah, Mona Massoud, Laurent Gaucher, Benoit de la Fourniere

**Affiliations:** 1 Department of Gynecology and Obstetrics, Hospices Civils de Lyon, Croix-Rousse Hospital, 103 Grande rue de la Croix Rousse, Lyon, 69001 France; 2 Obstetric Units, Child Couple University Hospital, University of Grenoble, Grenoble, F- 38700 France; 3 Obstetric research lab Department of Women-Mother-Child, Gynecology and Obstetrics Unit, Lausanne University Hospital (CHUV), Lausanne, Switzerland; 4 UZ Brussel, VUB, Brussels, Belgium; 5 Department of Maternal Fetal Medicine, Hospices Civils de Lyon, Femme Mère Enfant Hospital, 59 Boulevard Pinel, Bron, 69500 France; 6 Department of Obstetrics and Fetal Medicine, Lyon Sud Hospital Center, Lyon, France; 7 Geneva School of Health Sciences, HES-SO University of Applied Sciences and Arts, Western Switzerland 47 av. de Champel, Geneva, CH-1206 Switzerland; 8 Research on Healthcare Performance (RESHAPE), Université Claude Bernard Lyon 1, INSERM U1290, Lyon, F-69008 France; 9 LabTAU (INSERM 1032), Cours Albert Thomas, Lyon, France; 10 Laboratoire CarMeN - IRIS Team, INSERM U1060, INRA, Université Claude Bernard Lyon-1, INSA-Lyon, Univ-Lyon, Bron, 69500 France

**Keywords:** Macrosomia, Multicentric, Induction of labor, Cesarean section, Postpartum hemorrhage, France

## Abstract

**Introduction:**

Macrosomia, defined as a birth weight exceeding 4,000 grams, is an increasing concern in obstetric practice due to its associated delivery risks. Early induction of labor for large-for-gestational-age fetuses has been proposed to reduce shoulder dystocia and birth trauma, but the impact on maternal outcomes remains controversial.

**Methods:**

This retrospective multicenter study was conducted across three French university hospitals over a five-year period (2017–2023). Included were women with singleton term deliveries (≥37 weeks) and confirmed birth weight >4,000 g. Women with more than one previous cesarean, multiple pregnancy, breech presentation, or medical indications for elective cesarean were excluded.

Labor induction was performed according to local DAME-based protocols. Patients were admitted either the morning of or the day before induction. For a Bishop score <6, cervical ripening was proposed using prostaglandins, oral misoprostol, or a double-balloon catheter, with method selection based on patient preference. For a Bishop score ≥6, induction of contractions was initiated using prostaglandins.

The primary outcome was mode of delivery; secondary outcomes included postpartum hemorrhage (PPH), perineal injury, and neonatal morbidity (Apgar score, cord pH, shoulder dystocia). Analyses were adjusted for maternal age, pre-pregnancy BMI, parity, diabetes, and birth weight.

**Results:**

Among 76,375 deliveries, 4,300 met inclusion criteria (1,794 inductions and 2,496 spontaneous labors). Induction was associated with higher cesarean rates (45% vs. 10%; adjusted OR 2.43 [1.42–4.17] at 38 weeks and 1.85 [1.24–2.78] at 39 weeks) and increased PPH (27% vs. 15%; adjusted OR 1.82 [1.17–2.84] and 1.51 [1.08–2.11], respectively). Multiparity was protective, while each BMI unit increased cesarean risk by 6%. No significant differences were observed in Apgar <7, shoulder dystocia, or neonatal pH. Results were consistent in sensitivity analyses using ≥90th and ≥95th percentile thresholds.

**Conclusions:**

In confirmed macrosomia, labor induction was associated with increased cesarean delivery and postpartum hemorrhage without neonatal benefit. These findings highlight the need for cautious, individualized decision-making and improved accuracy of prenatal fetal weight estimation to reduce unnecessary inductions.

**Trial registration:**

The study was declared to CNIL: 23-5317 and registered on ClinicalTrials.gov: NCT06198881. (Registration date: January 10, 2024).

**Supplementary Information:**

The online version contains supplementary material available at 10.1186/s12884-026-09090-5.

## Condensation

Labor induction in confirmed macrosomia increases cesarean and adverse maternal risks versus spontaneous labor.

## Introduction

Macrosomia, defined as a birth weight exceeding 4,000 g, has become a growing concern in obstetric practice due to its implications for both maternal and neonatal outcomes [1]. Its prevalence continues to rise, affecting approximately 10% of pregnancies worldwide and 8.7% in France in 2022 [2]. Macrosomia is associated with a higher likelihood of cesarean section and multiple short- and long-term complications, including postpartum hemorrhage, shoulder dystocia, brachial plexus injury, and long-term metabolic consequences such as obesity, type 2 diabetes, and metabolic syndrome later in life [3–5].

The management of pregnancies complicated by suspected fetal macrosomia remains controversial. Early induction of labor for large-for-gestational-age fetuses has been proposed to reduce birth trauma and shoulder dystocia [6, 7]. The DAME trial demonstrated that induction between 37 + 0 and 38 + 6 weeks reduced both birth weight and shoulder dystocia while increasing the likelihood of spontaneous vaginal delivery [8]. However, other studies have shown conflicting results, reporting no significant difference in cesarean or neonatal outcomes between induction and expectant management [9, 10].

The ARRIVE trial, although often cited in discussions on elective induction, evaluated low-risk nulliparous women and did not specifically address suspected macrosomia [11]. Therefore, extrapolating its findings to large-for-date fetuses is not appropriate. Moreover, subsequent observational studies following ARRIVE, such as those by Gilroy et al. and Fineberg et al., have shown variable results regarding cesarean rates and maternal outcomes [12, 13]. Rather than focusing on elective induction in general, the current debate should emphasize the accuracy of prenatal estimation of fetal weight, which remains limited even with standardized formulas such as Hadlock’s [14, 15]. Diagnostic uncertainty may lead to unnecessary inductions for fetuses wrongly suspected as macrosomic, or missed cases of true macrosomia, both of which have clinical consequences.

National and local protocols vary widely in their management of suspected macrosomia, with thresholds for recommending induction ranging from an estimated fetal weight (EFW) of ≥ 4,000 g, particularly in the presence of maternal diabetes [16] These variations highlight the lack of consensus and the need for real-world data to support clinical decision-making.

In this context, our study aimed to compare obstetric outcomes among confirmed cases of fetal macrosomia across three tertiary centers. The primary objective was to describe the modes of delivery in confirmed macrosomic infants. Secondary objectives included comparing maternal and neonatal outcomes between induced labors for *suspected* macrosomia and spontaneous labors with *unexpected* macrosomia. Finally, we compared induced labors with expectant management and conducted subgroup analyses by parity, diabetes status, and trial of labor after cesarean (TOLAC).

## Material & methods

### Study design and setting

This retrospective multicenter observational study was conducted across three French university hospitals belonging to the same regional perinatal network. Data were collected over a five-year period, from January 1, 2017, to December 31, 2023.

### Study population

Eligible participants were women who delivered a singleton at term (≥ 37 weeks of gestation) with a birth weight > 4,000 g and a complete electronic medical record. Women were excluded if they had preterm birth, breech presentation, multiple pregnancy, more than one previous cesarean section, or a clear medical indication for elective cesarean section.

### Definition of suspected and confirmed macrosomia

Suspected macrosomia was defined before delivery in the medical record according to the DAME criteria (Table 1), including an estimated fetal weight (EFW) ≥97th percentile on ultrasound, maternal diabetes, or clinical suspicion based on uterine height or palpation. Confirmed macrosomia referred to an actual birth weight > 4,000 g.

**Table 1:** DAME trigger criteria [4]

Week of amenorrhea	Uterine height (cm)	Ultrasound estimation (g)
36	*≥* 34	*≥* 3500
37	*≥* 34	*≥* 3700
38	*≥* 35	*≥* 3900

Women were categorized into three comparison groups:

1. Induced labor for suspected macrosomia;
2. Spontaneous labor with unexpected macrosomia;
3. Expectant management, including deliveries occurring after the week when induction was performed.

**Fig. 1:**
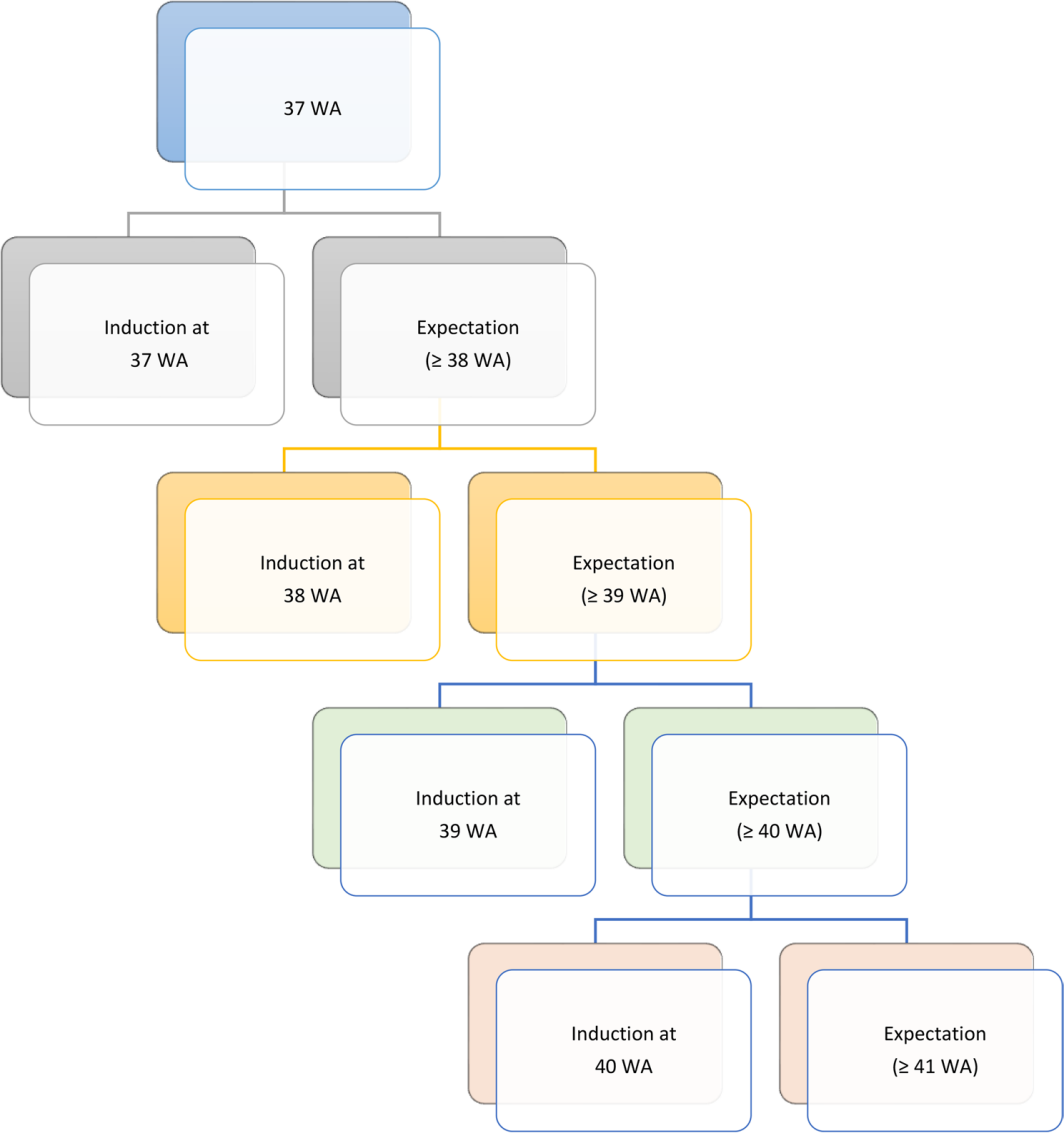
Flowchart of study population and comparison design. Each induction cohort (e.g., at 38 weeks of gestation) was compared with all deliveries occurring at a later gestational age (spontaneous or induced), excluding earlier inductions. Prophylactic cesarean sections performed before labor onset were excluded from the induction group but reported separately for transparency

This classification corresponds to Fig. 1, where each weekly induction cohort (e.g., 38 weeks) was compared with deliveries at a later gestational age, regardless of whether these subsequent births occurred spontaneously or following induction.

### Labor induction protocol

Patients were admitted either on the morning of the planned induction or the preceding evening, depending on bed availability and labor room capacity. Cervical status was assessed upon admission using the Bishop score.

- When the Bishop score was < 6, cervical ripening was proposed, with three available methods — vaginal prostaglandins, oral misoprostol, or double-balloon catheter. Each method was explained to the patient, who then selected her preferred option.
- When the Bishop score was ≥ 6, induction of uterine contractions was performed directly using prostaglandins.

These practices were consistent across centers and in accordance with French national obstetric recommendations. No inductions were performed using amniotomy alone.

### Data collection

Data were extracted by trained obstetricians and research midwives from standardized electronic medical records and verified manually for completeness. In the event of discrepancies, the original obstetric charts were reviewed for confirmation. Maternal data included maternal age, pre-pregnancy body mass index (BMI), parity, history of cesarean section, and diabetes type (type 1, type 2, or gestational) [15]. Maternal body mass index (BMI) was calculated from pre-pregnancy weight recorded at the first prenatal visit. Neonatal data included sex, birth weight, Apgar score at 5 min, umbilical arterial pH (< 7.10 considered low), shoulder dystocia, and neonatal complications [16].

The term “prophylactic cesarean” referred to cesarean deliveries performed before the onset of labor, mostly for suspected extreme macrosomia or significant maternal comorbidities [17]. These were excluded from the induction group but reported separately in the flowchart for transparency.

### Statistical analysis

Analyses were conducted using R Studio (v4.2) and Stata (v17) [18]. Continuous variables were expressed as means with standard deviations (SD), and categorical variables as frequencies and percentages. Differences between groups were analyzed using Student’s *t* test, Mann–Whitney *U* test, or chi-square test as appropriate [19].

Crude and adjusted odds ratios (ORs, aORs) were calculated using multivariable logistic regression models. Adjustments included maternal age, pre-pregnancy BMI, parity, diabetes status, and birth weight. These variables were selected based on their known association with both induction decisions and perinatal outcomes.

Subgroup analyses examined the influence of parity (primiparous vs. multiparous), diabetes, and trial of labor after cesarean (TOLAC) on delivery mode and maternal outcomes. Sensitivity analyses were conducted using percentile-based definitions of macrosomia (≥ 90th and ≥ 95th percentile). All tests were two-sided with a significance threshold of *p* < 0.05. Missing data accounted for < 2% of variables and were handled using complete-case analysis.

## Results

During the study period, a total of 76,375 women delivered in the three participating hospitals. Among these, 4,300 had a birth weight greater than 4,000 g and met inclusion criteria. Ten records were excluded due to duplication or implausible birth weights.

### Study flow

**Fig. 2:**
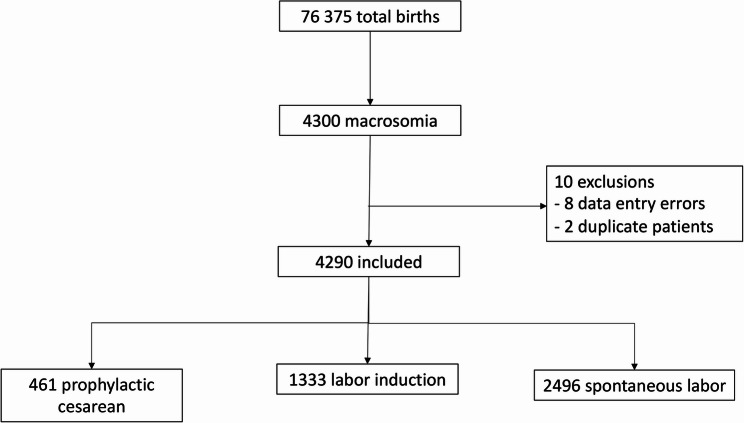
Flowchart of study participants

The study flowchart is shown in Fig. 2. Among the 4,761 women initially identified with suspected or confirmed macrosomia, 461 underwent prophylactic (elective) cesarean delivery prior to labor onset for suspected extreme macrosomia or significant maternal comorbidities; these were excluded from the induction group but reported separately for transparency. The remaining 4,300 deliveries were analyzed, comprising 1,794 inductions and 2,496 spontaneous labors.

### Induction methods

Among women in the induction group, 674 underwent cervical ripening (465 vaginal prostaglandins, 134 oral misoprostol, 75 double-balloon catheters), and 617 received oxytocin following cervical ripening or as a direct induction method.

### Maternal characteristics

Baseline characteristics were largely comparable between the two groups (Table 2). Women in the induction group had higher mean BMI and a greater prevalence of gestational diabetes (17% vs. 7%) compared with those in the spontaneous labor group. The proportion of primiparous women was higher in the induction group (47% vs. 31%). Mean gestational age at delivery was slightly lower among induced women (40 + 2 weeks vs. 40 + 4 weeks).

**Table 2:** Cohort Characteristics

Characteristics	Spontaneous Labor (*n* = 2496)	Induction of Labor (*n* = 1794)	OR/SMD (95% CI)
Age (years, mean ± SD)	31.8 ± 4.6	32.0 ± 5.5	0.25 (0.18–0.30)
Pre-pregnancy BMI (kg/m², mean ± SD)	24.9 ± 5.0	27.1 ± 6.3	0.32 (0.26–0.38)
Obesity (BMI ≥ 30), n (%)	463 (19)	511 (28)	0.41 (0.35–0.48)
Severe obesity (BMI ≥ 35), n (%)	156 (6)	203 (11)	0.42 (0.35–0.49)
Gestational diabetes, n (%)	176 (7)	301 (17)	0.38 (0.31–0.46)
Primiparous, n (%)	774 (31)	845 (47)	0.52 (0.45–0.59)
Multiparous, n (%)	1722 (69)	949 (53)	0.51 (0.44–0.57)
Gestational age at birth (weeks, mean ± SD)	40 + 4 ± 0.9	40 + 2 ± 1.3	0.12 (0.06–0.18)

*BMI* Body Mass Index, *SMD* Standardized Mean Difference, *OR* Odds Ratio, *95% CI* 95% confidence interval

To improve clinical interpretation, additional information is presented in Table 2 on parity distribution (primiparous vs. multiparous) and obesity categories (BMI ≥ 30 and ≥ 35 kg/m²).

A small subset of cases (*n* = 186) was initially recorded as having more than one prior cesarean section; these were identified as coding errors and excluded from adjusted analyses.

### Mode of delivery

Cesarean delivery was significantly more frequent in the induction group (45% vs. 10%; crude OR 4.46, 95% CI 3.9–5.1).

When stratified by gestational week (Table 3), the adjusted odds of cesarean were highest for inductions at 38 weeks (aOR 2.43, 95% CI 1.42–4.17) and remained elevated at 39 weeks (aOR 1.85, 95% CI 1.24–2.78). Multivariable regression confirmed that multiparity was protective (aOR < 1), and each BMI unit increase was associated with a 6% higher cesarean risk, this effect was similar in both the induction and spontaneous labor groups. (Table 4)

**Table 3:** Frequency and odds ratio of cesarean delivery and postpartum hemorrhage, comparing women who underwent labor induction at each gestational week with women delivered at a later gestational age, either following spontaneous labor or induction

Week of Induction	Cesarean Rate, *n* (%)	Crude OR (95% CI)	Adjusted* OR (95% CI)	PPH Rate, *n* (%)	Adjusted* OR (95% CI)
37	5 (35%)	3.0 (0.99–8.90)	3.03 (0.72–12.76)	4 (29%)	1.17 (0.30–4.51)
38	33 (28%)	2.12 (1.41–3.20)	2.43 (1.42–4.17)	31 (26%)	1.82 (1.17–2.84)
39	57 (23%)	1.63 (1.20–2.22)	1.85 (1.24–2.78)	57 (23%)	1.51 (1.08–2.11)
40	46 (24%)	1.28 (0.91–1.82)	1.03 (0.65–1.62)	46 (23%)	1.32 (0.91–1.92)

*OR* odds ratio, *95% CI* 95% confidence interval, *PPH* postpartum hemorrhage

* WA = week of amenorrhea; Odds Ratio adjusted for parity (primi or multiparity), birth weight, maternal age, maternal BMI, maternal height, previous CS

**Table 4:** Post-Delivery Maternal and Fetal Outcomes

Characteristics	Spontaneous Labor *n* = 2496	Induction Labor *n* = 1794	OR/SMD*
Mode of Delivery (*n*, %)			
Spontaneous vaginal birth	1947 (78)	795 (44)	4.46 (3.9–5.1)
Instrumental	292 (12)	190 (11)	
Cesarean section	257 (10)	809 (45)	
Blood loss (mL, mean ± SD)	292 ± 323	441 **±** 445	0.39 (0.33–0.46)*
PPH (n, %)			2.18 (1.87–2.54)
No	2131 (85)	1306 (73)	
Moderate (500–1000 mL)	277 (11)	330 (18)	
Severe (> 1000 mL)	88 (4)	158 (9)	
Perineum (n, %)			
Intact	1187 (48)	1207 (68)	0.44 (0.38–0.50)
Uncomplicated tear	1225 (49)	547 (30)	
OASIS	84 (3)	40 (2)	
APGAR score < 7 at 5 min (n, %)	31 (1)	37 (2)	1.67 (1.0.4–2.71)
Umbilical arterial pH (mean ± SD)	7.26 **±** 0.07	7.25 **±** 0.08	/
Birthweight (mean, SD)	4264 (1403)	4280 (1020)	0.03 (-0.03–0.08)*
Dystocia (n, %)	88 (4)	50 (3)	1.27 (0.89–1.81)
Neonatal death (n, %)	1 (0)	1 (0)	0.72 (0.03–18.12)

*PPH* postpartum hemorrhage, *OASIS* Obstetric Anal Sphincter Injury, *SD* standard deviation, *PhA* arterial pH, *OR* odds ratio, *SMD* Standardized Mean Difference, *95% CI* 95% confidence interval

* = SMD

### Maternal outcomes

Postpartum hemorrhage (PPH) occurred in 27% of induced labors compared with 15% in spontaneous labors. Among induced women, 18% experienced moderate PPH and 9% severe PPH, versus 11% and 4% respectively in spontaneous labor. PPH was defined according to French national guidelines as moderate (500–1000 mL) and severe (> 1000 mL) blood loss.

Adjusted analyses confirmed significantly higher risks at both 38 weeks (aOR 1.82, 95% CI 1.17–2.84) and 39 weeks (aOR 1.51, 95% CI 1.08–2.11) for induction compared with expectant management.

### Neonatal outcomes

Neonatal outcomes were comparable between groups. There were no significant differences in 5-minute Apgar score < 7 (1–2%), low umbilical arterial pH < 7.10, or shoulder dystocia (4% vs. 3%, OR 1.27, 95% CI 0.89–1.81). One neonatal death occurred in each group.

### Sensitivity and subgroup analyses

Sensitivity analyses using percentile-based definitions (≥ 90th and ≥ 95th) confirmed the robustness of the main findings. Subgroup analyses revealed that the increased risk of cesarean and PPH was particularly pronounced among primiparous and diabetic women, as well as among those undergoing TOLAC.

## Discussion

In this large multicenter real-life cohort, induction of labor for suspected macrosomia was associated with significantly higher rates of cesarean delivery and postpartum hemorrhage compared with spontaneous labor, without any observed neonatal benefit. These associations persisted after adjustment for confounders and in sensitivity analyses, and were especially pronounced in primiparous, diabetic, and TOLAC subgroups [1–3].

### Comparison with previous studies

Our findings contrast with those of the DAME randomized trial, which reported reduced shoulder dystocia and increased spontaneous vaginal deliveries after early induction between 37 and 38 weeks [8]. This discrepancy likely reflects the difference between controlled randomized settings and real-world practice, where inductions are often performed for women with less favorable obstetric profiles or additional comorbidities [9, 10]. Consequently, the increased cesarean rate in our study may partly reflect clinical selection bias rather than a direct causal effect of induction itself.

The ARRIVE trial, frequently cited in discussions on elective induction, focused on low-risk nulliparous women and was not designed to address suspected macrosomia [11]. Subsequent observational data, including those by Gilroy et al. and Fineberg et al., have reported variable findings, with some showing higher and others lower cesarean rates following induction [12, 13]. Together, these inconsistencies emphasize that induction outcomes are highly dependent on case selection, cervical status, and local management protocols [14].

### Interpretation of results

Our results suggest that, in the setting of confirmed macrosomia, the principal risks associated with induction are maternal rather than neonatal. The significantly higher rates of cesarean and postpartum hemorrhage observed in our study may reflect both mechanical and physiological challenges associated with larger fetuses and prolonged labor following unsuccessful induction [3, 15].

Inductions performed before 39 weeks appeared particularly associated with cesarean delivery. This may be related to lower cervical favorability at earlier gestational ages or to earlier intervention in more complex pregnancies [16]. The inclusion of a detailed induction protocol in our methods clarifies that cervical ripening was individualized and involved shared decision-making, reflecting real-world French obstetric practice.

Importantly, neonatal outcomes such as Apgar score, cord pH, and shoulder dystocia were similar between groups, consistent with other large observational studies [9, 12, 17]. This suggests that induction does not reduce mechanical complications, even in large fetuses, and that routine induction for suspected macrosomia may not offer neonatal advantages [18, 19].

### Clinical implications

Our findings underscore the need for individualized decision-making in the management of suspected macrosomia. Induction may still be justified for specific indications such as poorly controlled diabetes or maternal anxiety, but not solely based on estimated fetal weight [20]. The higher risk of cesarean section and postpartum hemorrhage observed, especially in primiparous and diabetic women, suggests that clinicians should discuss these risks explicitly during prenatal counseling [21, 22].

### Strengths and limitations

The main strengths of this study include its large multicenter design, standardized data collection, and exclusion of women with more than one previous cesarean or clear medical indications for elective cesarean, thereby reducing selection bias [23]. Subgroup and sensitivity analyses across multiple macrosomia definitions (≥ 4000 g, ≥ 90th, ≥ 95th percentile) confirmed the robustness of our findings [24].

However, several limitations must be acknowledged. The retrospective design precludes causal inference, and residual confounding cannot be excluded [25]. The study focused on confirmed macrosomia, excluding false-positive prenatal diagnoses (EFW > 4000 g but birth weight < 4000 g), limiting generalizability to prenatal counseling [26]. Moreover, because only a small proportion of infants had a birth weight > 4500 g, separate analyses were not performed due to limited statistical power. This limitation has been acknowledged.

Nevertheless, this approach ensured a more objective evaluation of outcomes among truly large infants.

Finally, although all centers used Hadlock’s formula for estimated fetal weight, intra- and inter-observer variability and the inherent limitations of ultrasound estimation near term remain important sources of diagnostic uncertainty [27, 28].

### Future perspectives

Future prospective studies should aim to integrate maternal and fetal risk factors, refine fetal weight estimation methods—potentially using three-dimensional ultrasound or MRI—and explore optimal timing and methods of induction in pregnancies complicated by suspected macrosomia [29, 30]. Multicenter registries may also help evaluate the long-term maternal and neonatal outcomes following induction for suspected macrosomia.

## Conclusion

In this large multicenter retrospective study of confirmed fetal macrosomia, induction of labor was associated with a higher risk of cesarean delivery and postpartum hemorrhage compared with spontaneous labor, without any improvement in neonatal outcomes. These results persisted after adjustment for maternal and obstetric factors and were consistent across multiple sensitivity analyses.

The findings suggest that routine induction for suspected macrosomia should be approached with caution and replaced by individualized, evidence-based decision-making that considers maternal characteristics, cervical favorability, and local resources.

Improving the accuracy of prenatal fetal weight estimation remains a key priority to avoid unnecessary inductions and their associated risks. Future prospective research should focus on refining diagnostic criteria, evaluating higher weight thresholds (the 95th percentile), and identifying maternal subgroups that might truly benefit from early delivery.

## Supplementary Information

Supplementary Material 1.

## Data Availability

The datasets used and/or analyzed during the current study are available from the corresponding author on reasonable request.
