# Supplementary material for: Mode of delivery for confirmed macrosomia: a real-life multicentric observational study
Source: BMC Pregnancy Childbirth. 2026 Apr 27;26:640. doi: 10.1186/s12884-026-09090-5 (PMC13255393; doi:10.1186/s12884-026-09090-5)
Supplement: Supplementary file 1 — Supplementary Material 1. [file 12884_2026_9090_MOESM1_ESM.docx]

**Delivery Modalities for Confirmed Macrosomia: A Real-Life Multicentric Descriptive Study - SUPPLEMENTARY**

Table 1: Cohort Characteristics – Labor induction

| **Caracterisitics** | Total | Medical  n = 1333 | PC  n = 461 | OR / SMD |
| --- | --- | --- | --- | --- |
| **Age (years, mean, SD)** | 32.5 (5) | 32.1 (5) | 33.7 (4.8) | 0.3 (0.22 – 0.43) |
| **BMI (Kg/m2, mean, SD)** | 27.1 (6.3) | 26.5 (6) | 29.0 (6.9) | 0.4 (0.29-0.51) |
| **Maternal weight (kg, mean, SD)** | 75.2 (18.1) | 73.7 (17.4) | 79.5 (19.1) | 0.32 (0.22-0.43) |
| **Gravidity (mean, SD)** | 2.7 (1.7) | 2.6 (1.6) | 3.1 (1.7) | 0.3 (0.2-0.4) |
| **Parity (mean, SD)** | 2.0 (1.2) | 2.0 (1.2) | 2.4 (1.1) | 0.07 (0.03-0.17) |
| **Scarred uterus (n, %)** |  |  |  | 0.05 (0.04-0.06) |
| No | 1275 (71) | 1159 (87) | 116 (25) |  |
| One CS | 375 (21) | 160 (12) | 215 (47) |  |
| More than 1 CS | 144 (8) | 14 (1) | 130 (28) |  |
|  |  |  |  |  |
| **Gestational diabetes (n, %)** | 301 (17) | 195 (15) | 106 (23) | 0.57 (0.44-0.75) |
| **Gestational age at birth**  **WA (mean, SD)** | 40+2 (9) | 40+6 (8) | 39+2 (8) | 1.4 (1.26-1.49) |

*BMI = body mass index in kg/m^2; SD = standard deviation; CS = cesarean section; PC = prophylactic cesarean; OR = odds ratio; 95% CI = 95% confidence interval; SMD = Standardized Mean Difference*

Table 2: Post-Delivery Maternal and Fetal Outcomes for Induction Labor

| **Characteristics** | **Total** | **Medical**  n = 1333 | **PC**  n = 461 | **OR/SMD** |
| --- | --- | --- | --- | --- |
| **Mode of Delivery (n, %)** |  |  |  |  |
| Spontaneous vaginal birth | 795 (44) | 795 (60) | 0 (0) |  |
| Instrumental | 190 (11) | 190 (14) | 0 (0) |  |
| Cesarean section | 809 (45) | 347 (26) | 461 (100) |  |
| **Blood loss (mean, SD)** | 441 [445] | 406 [455] | 544 [399] | 0.3 (0.2-0.4) |
| **PPH (n, %)** |  |  |  | 0.46 (0.37-0.58) |
| No | 1306 (73) | 1026 (77) | 280 (61) |  |
| Moderate | 313 (18) | 196 (14) | 135 (29) |  |
| Severe | 158 (9) | 111 (8) | 46 (10) |  |
| **Perineum (n, %)** |  |  |  | / |
| Intact | 1207 (68) | 746 (56) | 461 (100) |  |
| Simple tear | 547 (30) | 547 (41) | 0 (0) |  |
| LOSA | 40 (2) | 40 (3) | 0 (0) |  |
| **Arterial cord blood pH (mean, SD)** | 9.8 [0.9] | 9.8 [0.9] | 9.8 [0.7] | / |
| **Arterial cord blood pH (mean, SD)** | 7.25 [0.08] | 7.25 [0.08] | 7.25 [0.7] | / |
| **Birthweight (mean, SD)** | 4280 [1020] | 4268 [1173] | 4314 [288] | 0.2 (0.1-0.3) |
| **Dystocia (n, %)** | 50 (3) | 50 (4) | 0 (0) | / |
| **Neonatal deaths (n, %)** | 1 (0) | 1 (0) | 0 (0) | / |

*PC = prophylactic cesarean ; PPH = postpartum hemorrhage; SD = standard deviation;; PhA = arterial pH; PhV = venous pH; OR = odds ratio; 95% CI = 95% confidence interval ; SMD = Standardized Mean Difference*

Table 4: Frequency and odds ratio (both crude and adjusted for risk factors) of cesarean delivery and postpartum hemorrhage among women who underwent labor induction at each gestational week, or delivered at a later gestational age, either through spontaneous labor or induction.

|  | **N (%)** | **OR brut**  **(CI 95%)** | **Adjusted OR**  **(CI 95%)** |
| --- | --- | --- | --- |
| **Cesarean section** |  |  |  |
| induction at 37 WA | 5 (35) | 3.0 (0.99-8.90) | 3.03 (0.72-12.76) |
| Parity |  |  | 0.06 (0.05-0.08) |
| Fetal weight |  |  | 1.00 (1.00-1.00) |
| Maternal age |  |  | 1.63 (1.26-2.11) |
| BMI |  |  | 1.05 (1.03-1.07) |
| Maternal height |  |  | 0.92 (0.90-0.94) |
| Scarred uterus |  |  | 20.0 (14.87-27.0) |
| induction at 38 WA | 33 (28) | 2.12 (1.41-3.20) | 2.43 (1.42-4.17) |
| Parity |  |  | 0.06 (0.44-0.79) |
| Fetal weight |  |  | 1.0 (1.0-1.0) |
| Maternal age |  |  | 1.69 (1.30-2.20) |
| BMI |  |  | 1.05 (1.03-1.07) |
| Maternal height |  |  | 0.92 (0.91-0.94) |
| Scarred uterus |  |  | 22.30 (16.35-30.42) |
| induction at 39 WA | 57 (23) | 1.63 (1.20-2.22) | 1.85 (1.24-2.78) |
| Parity |  |  | 0.06 (0.04-0.78) |
| Fetal weight |  |  | 1.0 (1.0-1.0) |
| Maternal age |  |  | 1.60 (1.21-2.12) |
| BMI |  |  | 1.05 (1.03-1.07) |
| Maternal height |  |  | 0.92 (0.90-0.94) |
| Scarred uterus |  |  | 22.95 (16.34-32.22) |
| induction at 40 WA | 46 (24) | 1.28 (0.91-1.82) | 1.03 (0.65-1.62) |
| Parity |  |  | 0.56 (0.38-0.83) |
| Fetal weight |  |  | 1.0 (1.0-1.0) |
| Maternal age |  |  | 1.74 (1.23-2.45) |
| BMI |  |  | 1.04 (1.02-1.07) |
| Maternal height |  |  | 0.91 (0.89-0.94) |
| Scarred uterus |  |  | 30.44 (19.47-47.60) |
|  |  |  |  |
| **PPH** |  |  |  |
| induction at 37 WA | 4 (29) | 1.95 (0.61-6.23) | 1.17 (0.30-4.51) |
| Parity |  |  | 0.39 (0.32-0.47) |
| Fetal weight |  |  | 1.0 (1.0-1.0) |
| Maternal age |  |  | 1.19 (0.96-1.46) |
| BMI |  |  | 1.01 (0.99-1.03) |
| Maternal height |  |  | 0.98 (0.96-0.99) |
| Scarred uterus |  |  | 2.26 (1.79-2.84) |
| induction at 38 WA | 31 (26) | 1.75 (1.15-2.66) | 1.82 (1.17-2.84) |
| Parity |  |  | 0.37 (0.31-0.45) |
| Fetal weight |  |  | 1.0 (1.0-1.0) |
| Maternal age |  |  | 1.23 (0.99-1.52) |
| BMI |  |  | 1.01 (0.99-1.03) |
| Maternal height |  |  | 0.98 (0.96-0.99) |
| Scarred uterus |  |  | 2.31 (1.83-2.92) |
| induction at 39 WA | 57 (23) | 1.49 (1.09-2.02) | 1.51 (1.08-2.11) |
| Parity |  |  | 0.38 (0.31-0.47) |
| Fetal weight |  |  | 1.0 (1.0-1.0) |
| Maternal age |  |  | 1.23 (0.98-1.54) |
| BMI |  |  | 1.01 (0.99-1.03) |
| Maternal height |  |  | 0.98 (0.96-0.99) |
| Scarred uterus |  |  | 2.39 (1.86-3.08) |
| induction at 40 WA | 46 (23) | 1.40 (0.99-1.99) | 1.32 (0.91-1.92) |
| Parity |  |  | 0.37 (0.29-0.48) |
| Fetal weight |  |  | 1.0 (1.0-1.0) |
| Maternal age |  |  | 1.19 (0.90-1.59) |
| BMI |  |  | 1.01 (0.98-1.02) |
| Maternal height |  |  | 0.97 (0.95-0.99) |
| Scarred uterus |  |  | 2.14 (1.55-2.96) |

*OR = odds ratio; 95% CI = 95% confidence interval; BMI = body mass index in kg/m^2; PPH = postpartum hemorrhage*
